# Toxic Epidermal Necrolysis Secondary to Metronidazole

**DOI:** 10.7759/cureus.17101

**Published:** 2021-08-11

**Authors:** Junaid Ali, Mansoor Rahman, Ammar Ahmad, Zoia Khattak, Muhammad Asim Shahzad

**Affiliations:** 1 Internal Medicine, Khyber Teaching Hospital, Peshawar, PAK; 2 Internal Medicine, Lady Reading Hospital MTI, Peshawar, PAK; 3 Medicine, Khyber Medical College, Peshawar, PAK; 4 Orthopaedics and Trauma, Khyber Teaching Hospital, Peshawar, PAK; 5 Nephrology, Rush University Medical Center, Chicago, USA

**Keywords:** hypersensitivity reaction, metronidazole, drug side effect, toxic epidermal necrolysis, stevens-johnson syndrome

## Abstract

Hypersensitivity reactions occur when a host exhibits an inappropriate or exaggerated response to allergens. Stevens-Johnson syndrome (SJS) and toxic epidermal necrolysis (TEN) are examples of such exaggerated responses to various drugs or illnesses. Both conditions affect the skin and mucosal surfaces of the oral cavity, urethra, and conjunctiva to varying degrees. TEN involves more than 30% of the total body surface area as opposed to SJS with less than 10% involvement. Skin biopsy is considered the gold standard for diagnosis; however, obtaining appropriate clinical context at presentation with the history of a potential offending drug can help diagnose the condition in situations where skin biopsy is not feasible. Metronidazole has been rarely reported as the offending agent for TEN/SJS with only two previously reported cases in the literature. We present the third case of TEN secondary to metronidazole and discuss the potential mechanism of action of metronidazole along with its common side effects. Our case adds to the existing literature of this rare clinical presentation and highlights the importance of the judicious use of metronidazole in clinical practice.

## Introduction

Stevens-Johnson syndrome (SJS) and toxic epidermal necrolysis (TEN) are infrequent and serious hypersensitivity reactions affecting the skin and mucous membranes. By definition, SJS affects less than 10% of the total body surface area (TBSA) while TEN involves greater than 30% of the TBSA. SJS/TEN usually starts with fever, sore throat, and generalized body aches, progressing to painful desquamating rash. In adults, it is mostly seen as an idiopathic reaction following the use of a drug, whereas in the pediatric population, it is usually seen after viral or bacterial infection [1]. The affected areas include the face, neck, trunk, and proximal parts of extremities including palm and soles with relative sparing of the distal arms and legs. In addition, conjunctiva and mucosal surfaces of the oral cavity and genital tract are involved in more than 90% of patients [2], with peculiar hemorrhagic crusting of the lips and mouth [3]. Antibiotics, especially sulfonamides, anticonvulsants, and nonsteroidal anti-inflammatory drugs (NSAIDs) are the offending agents in the majority of cases of SJS/TEN [4]. Metronidazole leading to the development of SJS/TEN is rare. To our knowledge, only two similar cases have been reported in the literature [5]. Metronidazole is frequently used empirically, especially in developing countries where food-borne illnesses are prevalent. Our case highlights the importance of detailed history-taking as well as the cautious empirical use of metronidazole to avoid this potentially fatal side effect.

## Case presentation

A 35-year-old male with no known past medical history presented to the emergency department with complaints of three-day history of fever, sore throat, and a skin rash. He had developed acute-onset watery diarrhea one week before this presentation. Infection workup for diarrhea was unremarkable and he was empirically prescribed oral metronidazole 400 mg three times a day along with oral rehydration solution. Subsequently, diarrhea resolved in two days; however, four days later, he noticed flu-like symptoms, malaise, and fatigability. These symptoms were followed by an abrupt appearance of a maculopapular rash involving the torso and bilateral lower extremities prompting his visit to the emergency department. He denied the use of any other medications except for metronidazole. A review of other systems was unremarkable. On examination, he had a low-grade fever of 100.4°F, blood pressure of 105/65 mmHg, heart rate of 100 per minute, and respiratory rate of 18 breaths per minute, with an oxygen saturation of 98% on room air.

The patient was in obvious physical distress due to pain. Bilateral conjunctivae were infected. The oral cavity showed multiple ulcers on the tongue and buccal mucosa. The neck was supple with no cervical or axillary lymphadenopathy. Lungs were clear on auscultation bilaterally. He had tachycardia with normal S1 and S2. His abdomen was mildly tender in all four quadrants without hepatosplenomegaly, rigidity, or guarding. He was alert and oriented without any gross focal neurological deficits. Skin examination was remarkable for maculopapular rash involving the face, neck, torso, abdomen, back, perineal area, as well as the bilateral upper and lower extremities. There were areas of desquamation specifically involving the upper and lower back, left eyelid, and genitalia.

Laboratory investigations revealed the following results: hemoglobin 14 g/dL, white blood cell count 3,500/µL, platelets 129,000/µL, neutrophils 88%, lymphocytes 10%, eosinophils 1%, sodium 130 mol/L, potassium 4.89 mmol/L, chloride 92.1 mmol/L, blood urea nitrogen 58 mg/dL, serum creatinine 1.21 mg/dL, blood glucose 200 mg/dL, total bilirubin 0.45 mg/dL, alanine aminotransferase 41.2 U/L, alkaline phosphatase 67 U/L, and creatine kinase 674 U/L. Infectious workup for diarrhea done one week ago including stool for ova and parasites, viral polymerase chain reaction panel, stool leukocytes, and stool culture was negative. Further viral tests including hepatitis A antibody, hepatitis B surface antigen, hepatitis C antibody, and human immunodeficiency virus antibody were negative. The CT scans of the chest, abdomen, and pelvis did not identify any pathology.

**Figure 1:**
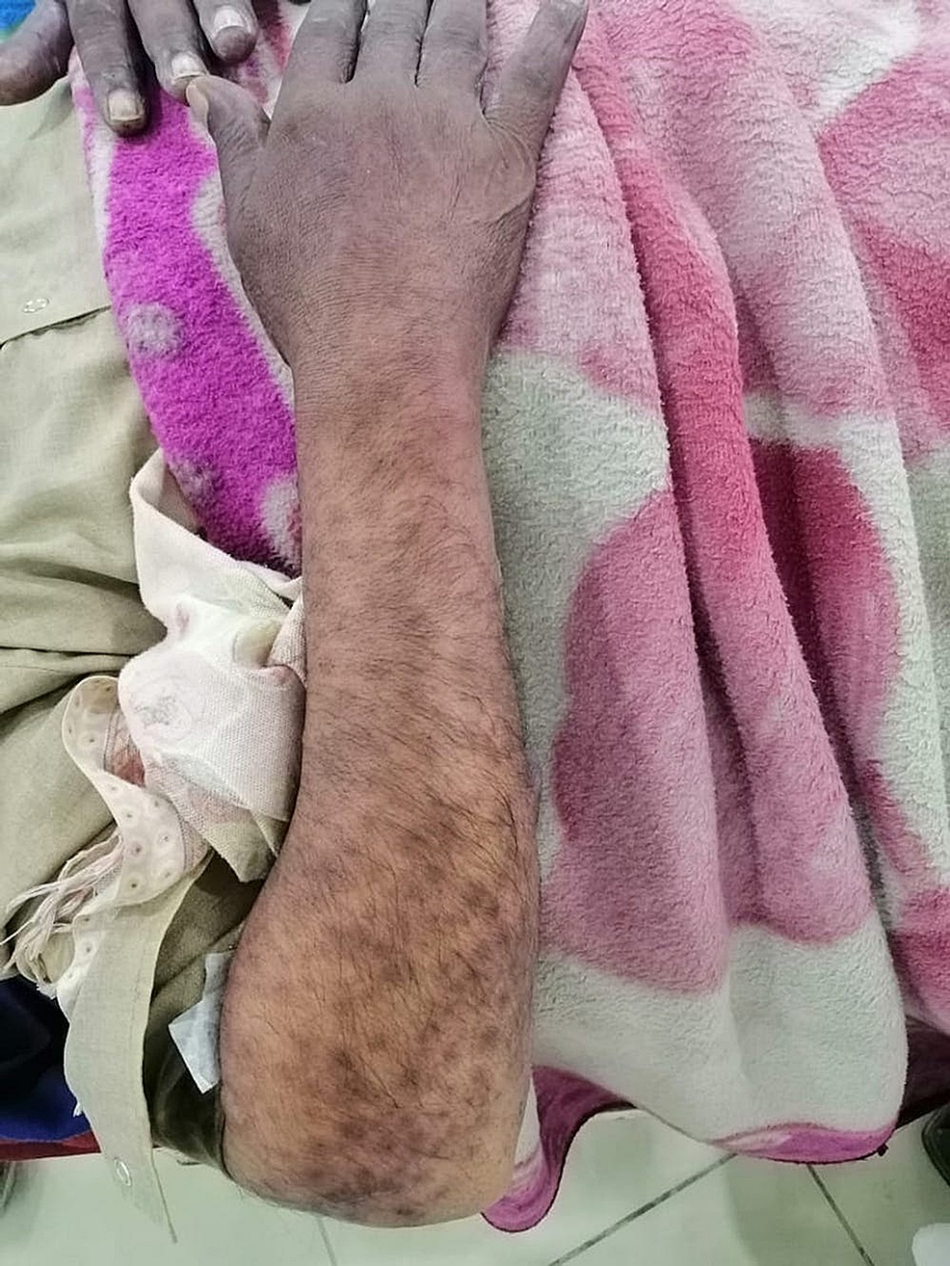
Right upper extremity with a characteristic macular and papular rash.

**Figure 2:**
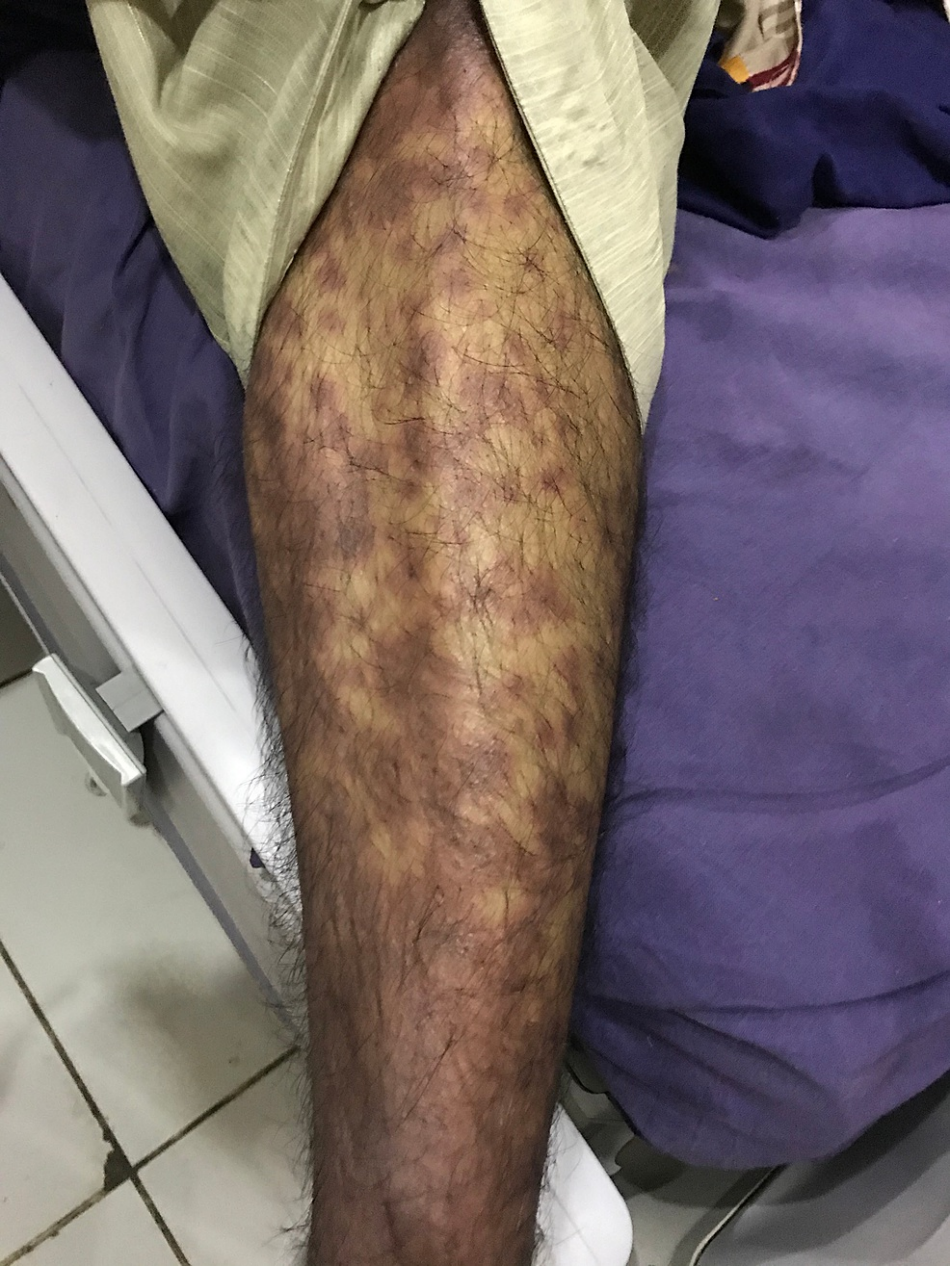
Left lower extremity with a characteristic macular and papular rash.

**Figure 3:**
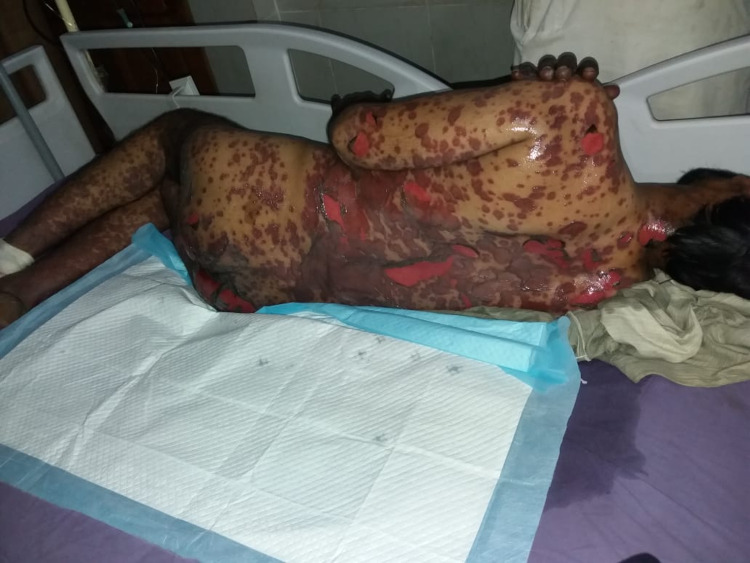
Bullous lesions on the back with positive Nikolsky sign.

**Figure 4:**
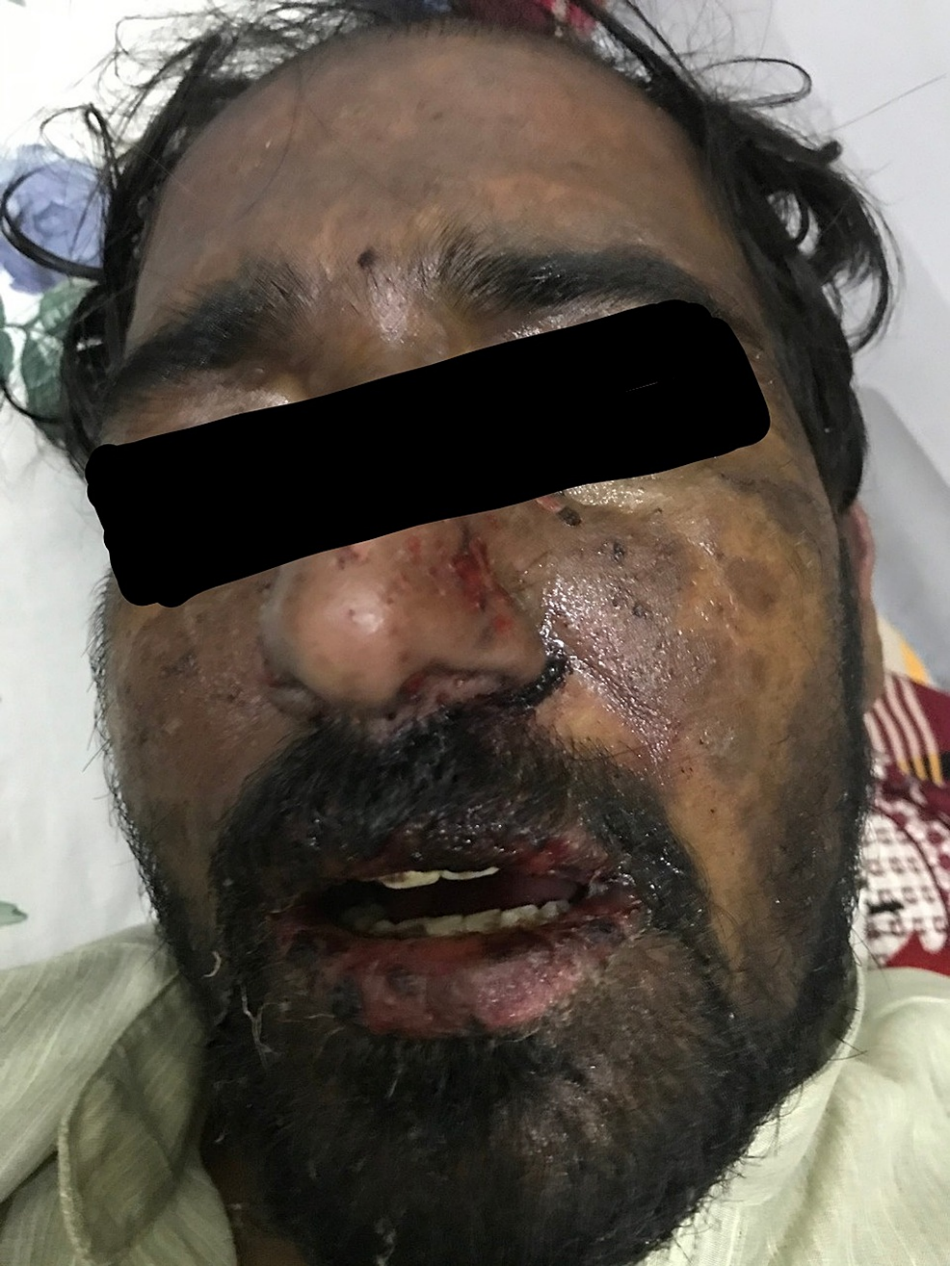
Mucosal ulcers of the oral cavity.

He was started on supportive therapy with aggressive intravenous fluids for volume replacement. Metronidazole was stopped and intravenous dexamethasone was started along with empirical broad-spectrum antibiotics, topical steroids, and emollients. Although the plan was to perform a skin biopsy, it was deferred due to the patient’s and his family’s refusal. His clinical condition continued to worsen with rapidly progressing desquamating skin and mucosal lesions despite aggressive supportive therapy. On the third day of hospitalization, he became hemodynamically unstable and was transferred to the intensive care unit (ICU) where he developed septic shock requiring vasopressor support. The patient’s condition worsened rapidly. Skin lesions (macules and papules) extended to the distal parts of the upper and lower extremities (Figures 1, 2), chest, upper and lower back, and genitalia. Lesions on the back transformed into bullae, which eventually sloughed off (Nikolsky sign) (Figure 3). He also developed mucosal ulcers in the oral cavity (Figure 4) and penile urethra. Based on the history and clinical examination, TEN caused by metronidazole was diagnosed. He did not respond to supportive therapy in the ICU and eventually developed cardiac arrest.

 

## Discussion

SJS and TEN are serious hypersensitivity reactions often precipitated by drugs such as sulfonamides, anticonvulsants, and NSAIDs. Although SJS and TEN were previously considered separate, they are now considered to be a part of a continuum, with SJS and TEN involving less than 10% and 30% of the TBSA, respectively. TEN is rare, acute, and can be potentially fatal. It often begins with fever and flu-like symptoms, followed within days by widespread blistering and mucosal involvement, which can include oral, urethral, and conjunctival mucosa. Although TEN is postulated to be drug-related, it is oftentimes difficult to prove the causation. An in-depth review of a patient’s history remains the best tool to identify a particular drug to be a trigger for SJS/TEN. The re-challenge test helps confirm the diagnosis but cannot be performed due to obvious ethical reasons and risk of exposure to offending agents with worsening of the clinical syndrome. A skin biopsy remains the gold standard to diagnose this condition. It can take up to one to three weeks for drug-induced SJS/TEN to occur, which is relatively quick with the re-administration of drugs. SJS/TEN is commonly caused by drugs such as sulphonamides, nevirapine, allopurinol, lamotrigine, aromatic anticonvulsants, oxicam, and other NSAIDs. Life-threatening reactions occur mostly in the case of drugs that have long half-lives [6]. Table 1 lists the commonly implicated classes of drugs in SJS/TEN along with specific examples.

**Table 1:** Drug classes and common examples of medications causing SJS/TEN. NSAID: nonsteroidal anti-inflammatory drugs; SJS: Stevens-Johnson syndrome; TEN: toxic epidermal necrolysis

Drug class	Common examples
Antibiotics	Cephalosporins, fluoroquinolones, penicillins, macrolides, vancomycin
Anticonvulsants	Carbamazepine, phenytoin, valproic acid, lamotrigine
Antihyperuricemic	Allopurinol
Antivirals	Nevirapine, abacavir
Antitubercular	Isoniazid, rifampin, ethambutol
Sulfonamides	Co-trimoxazole, sulphadiazine, sulphasalazine
NSAIDs	Piroxicam, aspirin, ibuprofen, diclofenac, naproxen, celecoxib

Metronidazole is a synthetic nitroimidazole-derived bactericidal agent widely used in the treatment of various anaerobic, protozoal, and parasitic infections. The precise mechanism of action of metronidazole is yet to be fully elucidated; however, unionized metronidazole is taken up readily by obligate anaerobes and reduced to an active intermediate product that breaks the DNA strands, thereby inhibiting DNA synthesis and cell growth. The characteristic feature of anaerobic bacteria converting metronidazole to nitroso-containing intermediate products via a reduction reaction makes metronidazole a treatment choice in anaerobic infections. The helical structure of DNA is disrupted and breaks are formed in the strands after the intermediate products covalently bind to it, inhibiting the synthesis of bacterial nucleic acid and ultimately resulting in cell death. Additionally, it plays a role as an antitrichomonal drug, a prodrug, an antibacterial drug, an antimicrobial agent, an antiparasitic agent, a xenobiotic, an environmental contaminant, and a radiosensitizing agent [7]. A range of adverse effects is associated with the use of metronidazole such as nausea, vomiting, diarrhea, dizziness, headache, metallic taste, pain in the abdomen, as well as weight loss, which occurs in more than 1% of patients treated with this agent. Allergic reactions in the form of skin rashes, pruritus, and fever along with glossitis, stomatitis, paresthesia, and dark urine are described as rare events. Thrombophlebitis also occurs in the case of intravenous administration of metronidazole [8]. The duration for the onset of symptoms can vary. During our exhaustive search of the literature, we found a case report where the patient developed SJS six hours after the first dose [5]. Another report by Piskin and Mekkes noted the development of symptoms 24 hours after the initiation of metronidazole [9]. With the use of the World Health Organization-Uppsala Monitoring Centre system for standardized case causality assessment [10] and Naranjo algorithm [11], with a Naranjo score of 6, TEN in our patient was likely related to the administration of metronidazole. Clinicians should discuss this rare side effect of metronidazole with all patients before its use. Judicious use of this drug can potentially avoid such catastrophic events in the future. In developing countries, prescription of antibiotics should be limited to licensed practitioners who are well aware of the side effects of the drug. By this case report, we would like to educate clinicians about this rare association of metronidazole and SJS/TEN.

## Conclusions

Metronidazole is a frequently used antibiotic for various infectious illnesses. Its empirical use is very common, especially in developing countries. Metronidazole can lead to severe hypersensitivity reactions such as SJS and TEN even at therapeutic dosages. Clinicians should judiciously use this drug and discuss this potential side effect with patients before use.
